# Adenomatoid odontogenic tumor associated with odontoma: a case report and critical review of the literature

**DOI:** 10.1186/1746-160X-9-20

**Published:** 2013-08-09

**Authors:** Ricardo Santiago Gomez, Wagner Henriques Castro, Carolina Cavaliéri Gomes, Adriano Mota Loyola

**Affiliations:** 1 Department of Oral Pathology, School of Dentistry, Universidade Federal de Minas Gerais, Belo Horizonte, Minas Gerais, Brazil; 2 Department of Pathology, Biological Sciences Institute, Universidade Federal de Minas Gerais, Belo Horizonte, Minas Gerais, Brazil; 3 Department of Oral Pathology, School of Dentistry, Universidade Federal de Uberlândia, Uberlândia, Minas Gerais, Brazil

## Abstract

We describe a case of adenomatoid odontogenic tumor (AOT) associated with odontoma occurring in the posterior mandible of a 32-year-old man. Although calcifications are commonly found in the AOT, the presence of rudimentary dental structures is a very rare phenomenon. Cases with similar aspects have been described as ameloblastic dentinoma, ameloblastic odontoma, adenoameloblastic odontoma and AOT associated with odontoma. After a careful analysis of the literature we describe the clinical aspects of this tumor. Further case reports and surveys of odontogenic tumors are necessary to define whether AOT associated with odontoma is a variant of AOT or a distinct clinicopathologic condition.

## 

Odontogenic tumors and hamartomas encompass a large variety of rare lesions that originate from odontogenic tissue and present with variable levels of differentiation. Determination of their precise nature (i.e., a hamartoma or a neoplasm) is difficult and sometimes inconclusive. This, in turn, makes it difficult to devise a nomenclature for this group of lesions.

In 1998, Allen and co-workers [1] described a rare odontogenic lesion showing a distinct proliferative component of epithelial cells arranged in cords, strands, swirls, and duct-like structures associated with a prominent dentin formation lined by ameloblast-like cells [1]. The prominence of dentin and adenomatoid structures in this lesion led the authors to describe it as an “adenomatoid dentinoma”. This lesion showed overlapping features of an adenomatoid odontogenic tumor (AOT) and an odontoma, with histological features similar to those reported by Dunlap et al., [2] and was described as an adenoameloblastic odontoma. Subsequently, different terms were used to describe similar lesions and, up to now, there is no consensus on to the nature, histopathological spectrum, and clinical significance of this lesion. Here, we report a similar case that exemplifies the odontogenic potential of an AOT and we present a critical analysis of the literature regarding this diagnosis.

## Case presentation

**Figure 1:**
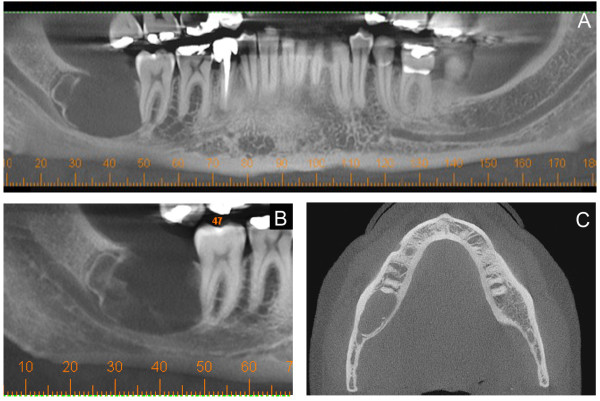
Computerized tomography scan in saggital (A and B) and axial (C) planes showing unilocular well defined hypodense mandibular tumor.

**Figure 2:**
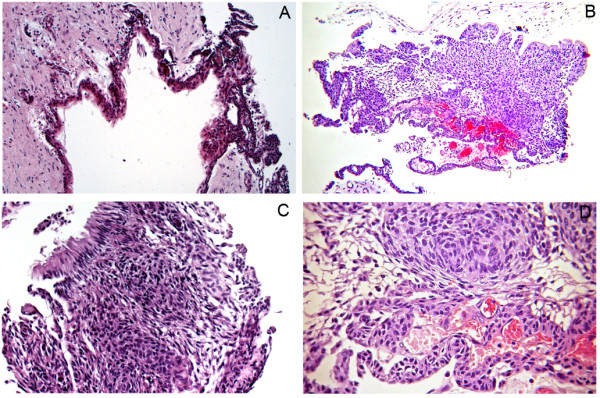
**Histological aspects of the epithelial component of the tumor.** Cystic cavity lined by flattened, stratified non-keratinized squamous epithelium **(A)**. Epithelial cords and strands forming swirls and nodules of fusiform cells and ribbons of ameloblast-like cells **(B**, **C** and **D)** (hematoxylin-eosin, original magnification X 200 and X 400).

**Figure 3:**
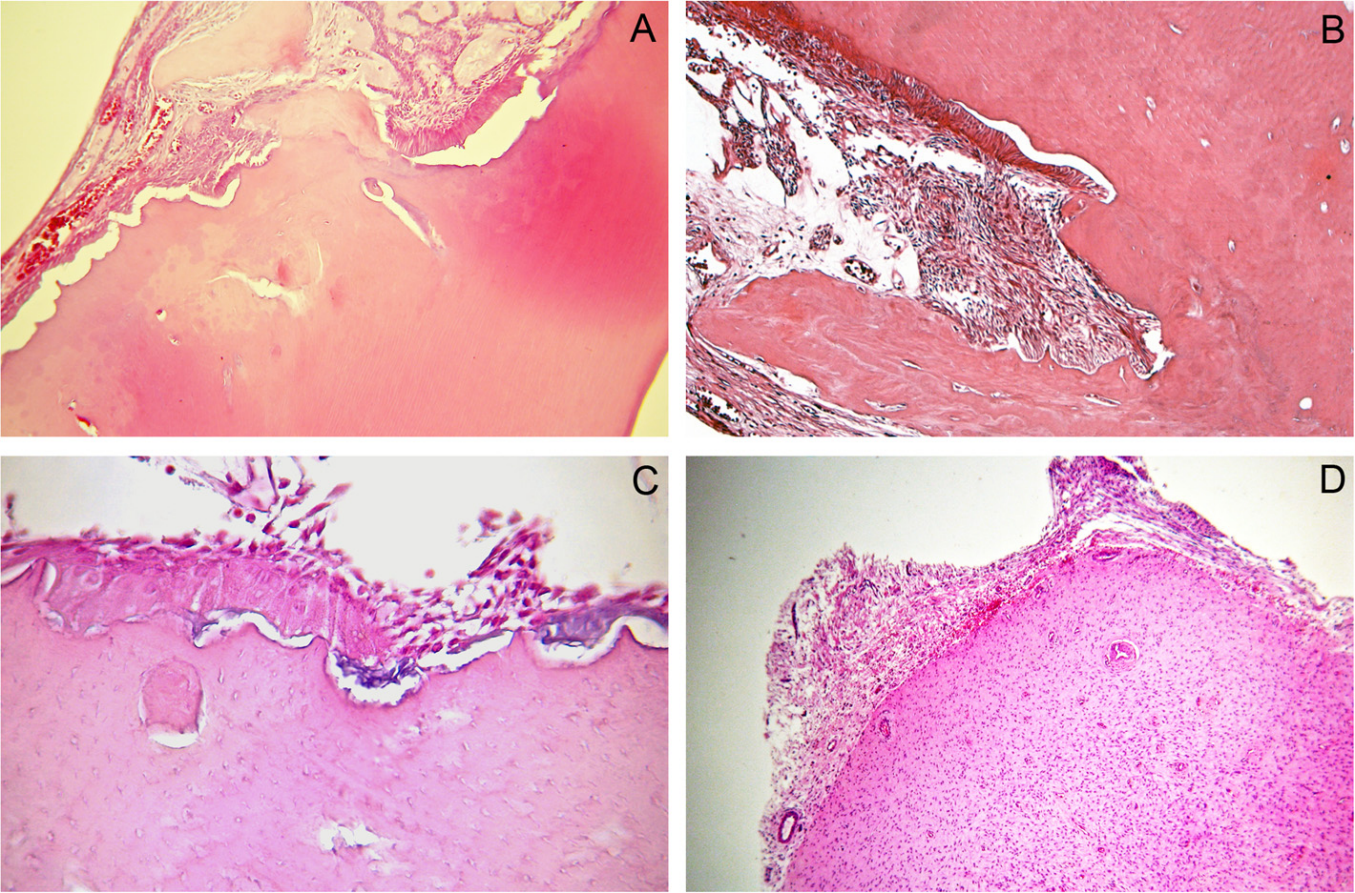
**Microscopic details of the odontoma component showing dentin deposition with lacunar borders (A) lined by ameloblast-like cells and sheets of odontogenic epithelial cells (B).** Thin basophilic layer consistent with enamel matrix deposit **(C)** and dental pulp tissue **(D)** were observed (hematoxylin-eosin, original magnification X 200 and X 400).

A 32-year-old man presented with an asymptomatic, well-delimitated, 2.6 x 2.1 x 1.3 cm unilocular hypodense tumor in the left posterior mandible (Figure 1). On examination of the oral cavity, a mild expansion of the retromolar trigone was observed. An incisional biopsy was performed, revealing a cystic capsule lined with stratified squamous, non-keratinized epithelium. These findings suggested a benign odontogenic cystic lesion. The lesion was completely enucleated and was found to contain a cystic capsule adhering to a thin hard tissue resembling a tooth. Microscopic observation showed a cystic cavity lined by flattened, stratified non-keratinized squamous epithelium. In some areas, we observed nodules, cords and strands in the epithelial lining forming swirls of fusiform cells and ribbons of ameloblast-like cells (Figure 2). We also observed numerous islands of odontogenic epithelium in the fibrous capsule and solid areas formed mainly by dentin with lacunar borders externally lined by ameloblast-like cells (Figure 2). Some areas had a thin layer with an appearance similar to that of an enamel matrix deposit (Figure 3C). The diagnosis was of AOT associated with odontoma. No recurrence was found upon an 8 months follow-up after enucleation of the lesion.

## Discussion

The present report describes a cystic lesion characterized histologically by the presence of an AOT associated with an odontoma. Previous reports of lesions with AOT-like epithelial structures and rudimentary dental formation have variously described them as adenoameloblastic odontomas [2], cystic complex compound odontomas [3], adenomatoid dentinomas [1,4], AOT associated with odontoma [5], and AOT [6]. One can conclude from the variable nomenclature used that no consensus has been reached regarding the precise nature and definition of histological characteristics of such lesions.

After extensive review of the literature on lesions with similar histological aspects, we retrieved those case reports showing overlapping features of AOT and odontoma or dental tissue presenting with rudimentary odontogenesis described under the following nomenclature: ameloblastic dentinoma, adenoameloblastic odontoma, AOT associated with odontoma and adenomatoid odontoma [1-7]. Only cases with published histological pictures of these findings were included. The clinical and radiographic features together with the clinical behavior of the AOT–odontoma variants reported in the literature are listed Table 1. This variant of AOT affects patients aged 4 to 46 years (mean 24.4 years). Five of the 11 patients were younger than 18 years. Instead of being located in the anterior maxilla, which is more typical of AOT [8], this odontoma-associated variant was mainly present in the posterior mandible (9 of 11 cases) and was located in the maxilla in only one case. Radiographically, the tumor typically results in a well-defined radiolucent image, sometimes exhibiting subtle calcifications or radiopaque deposits within the lesion [1,4]. A radiopaque image was noted in 1 case [6].

**Table 1:** Previous reports of adenomatoid odontogenic tumor (AOT) associated with odontoma

**Authors**	**Nomenclature**	**Gender**	**Age**	**Site**	**Radiographic features**	**Size (cm)**	**Treatment**	**Follow-up (Recurrencec**
Miles [3]	Cystic complex composite odontoma	M	18	Posterior mandible	Well-circumscribed radiolucent lesion-like dentigerous cyst	NA	Enucleation	NA
Dunlap & Fritzlen [2]	Adenoameloblastic odontoma	F	4	Posterior mandible	Well-defined radiolucent lesion	NA	Enucleation	NA
Tajima et al. [6]	AOT arising in an odontogenic cyst	M	15	Maxillary sinus	Well-defined radiopaque mass	4.0 × 4.0	Enucleation	5 years (No)
Allen et al. [1]	Adenomatoid dentinoma	M	37	Mandibular third molar region	Unilocular radiolucency	1.5 × 2	Enucleation	2 years (No)
	Adenomatoid dentinoma	M	29	Mandibular third molar region	Well-circumscribed radiolucency with subtle linear radiopacity within the lesion	0.75 × 0.75	Enucleation	8 years (No)
	Adenomatoid dentinoma	M	35	Left posterior mandible	Unilocular radiolucency	1 × 0.8	Curettage	3 years (No)
	Adenomatoid dentinoma	F	29	Mandibular third molar region	Radiolucency with subtle focal calcifications	2.0 × 2.75	Surgery, not specified	8 years (No)
Cudney et al. [7]	AOT associated with odontoma	M	13	Mandibular left canine	Pericoronal radiolucency with faint snowflake calcifications	1.4 × 0.7	Excisional biopsy	1 year (No) ^1^
Kemp et al. [4]	Adenomatoid dentinoma	M	46	Right mandibular body and ramus	Well-circumscribed with focal internal radiopacity	4.0 × 2.5	Enucleation and curettage	6 months (No)
Martínez et al. [5]	AOT concomitant with cystic complex odontoma	F	10	Left posterior mandible	Well-circumscribed radiolucency with radiopaque mass inside	3.0 × 1.5	Enucleation with posterior curettage	NA
Gomez et al. (present report)	AOT associated with odontoma	M	33	Left mandibular third molar region	Well-delimitated unilocular radiolucency	3.0 × 2.0	Enucleation	8 months (No)

NA: not available.

The epithelial component of AOT associated with odontoma exhibits nodules of cuboidal and fusiform cells forming nests (condensations) or rosette-like structures and a variable number of duct-like formations lined by cuboidal or columnar cells. Interconnecting strands and ribbons with two or more cells are present throughout the lesion. In our case we did not find duct-like structures typically reported in AOT. However, due to the overall morphologic aspect of the tumor, this diagnosis can be made without the presence of these structures [9]. The presence of tubular dentin, with or without a lining of ameloblast-like cells, and an amorphous and/or reticulated enamel matrix complete the histological features of the lesion. These characteristics are essentially identical to those of lesions previously identified as AOT, in which histological elements of odontogenesis were also described. Until a better definition is established in the literature, our opinion is that this group should be defined as AOT associated with odontoma.

AOT associated with odontoma should not be confused with adenomatoid odontogenic hamartoma (AOH). The AOH is characterized by the deposition of dentin in a ring-like pattern with odontoblasts, dental papilla, lined on the outer surface by enamel, ameloblasts and stellate reticulum-like cells [10,11]. Small duct-like structures are seen on the dental organ-like area and they are lined by flattened or cuboidal cells [10]. This finding is in contrast to AOT, in which ducts are lined by columnar ameloblast-like cells. In addition, no rosette-like structures are identified in AOH. It appears that these formations represent a morphological variation similar to that of the external epithelium of the dental organ. On the basis of this observation and in agreement with the opinion expressed by Kemp et al. [4] we believe that AOH simply represents a failed attempt at tooth development, more specifically the third molar, and could be classified as hamartomatous in nature. Takeda et al. [12] showed similar morphological variations in the odontogenic epithelium of compound odontomas, which are different from those present in AOT.

In the case we report, we found a cystic cavity lined by stratified squamous, non-keratinized epithelium. The same microscopic appearance was reported by Tajima et al. [6], indicating that odontogenic cysts should be included in the differential diagnosis of this tumor.

Although some AOT with odontoma are large lesions [4,6], their clinical behavior seems similar to conventional AOT. None of the cases showed recurrence, suggesting that the tumor can be treated conservatively.

## Conclusion

In the present report we review the literature and describe the clinical and histological aspects of AOT associated with odontoma. Although this tumor differs in some aspects to classic AOT, further case reports and surveys of odontogenic tumors are necessary to define whether it is a variant of this odontogenic tumor or a distinct clinicopathological condition.

### Consent

The authors declare that the patient had given consent for the case report to be published.

## Competing interest

The authors declare that they have no competing interests.

## Authors’ contribution

WHC performed surgery and drafted the manuscript. AML, RSG and CCG performed histopathological examination, conducted a review and analysis of the literature, and drafted the manuscript. All authors read and approved the final manuscript.

## References

[B1] Allen CM Neville BW Hammond HL Adenomatoid dentinoma. Report of four cases of an unusual odontogenic lesion Oral Surg Oral Med Oral Pathol Oral Radiol Endod 1998 86 3 313 317 10.1016/S1079-2104(98)90178-0 9768421

[B2] Dunlap CL Fritzlen TJ Cystic odontoma with concomitant adenoameloblastoma (adenoameloblastic odontoma) Oral Surg Oral Med Oral Pathol 1972 34 3 450 456 10.1016/0030-4220(72)90324-6 4505759

[B3] Miles AE A cystic complex composite odontome Proc R Soc Med 1951 44 1 51 55 14808227 10.1177/003591575104400111 PMC2081599

[B4] Kemp S Gallagher G Kabani S Todd R Adenomatoid dentinoma: case report and review of a rare odontogenic lesion J Oral Maxillofac Surg 2008 66 7 1489 1491 10.1016/j.joms.2007.09.009 18571036

[B5] Martinez A Mosqueda-Taylor A Marchesani FJ Brethauer U Spencer ML Adenomatoid odontogenic tumor concomitant with cystic complex odontoma: case report Oral Surg Oral Med Oral Pathol Oral Radiol Endod 2009 108 4 e25 e29 10.1016/j.tripleo.2009.06.024 19778732

[B6] Tajima Y Sakamoto E Yamamoto Y Odontogenic cyst giving rise to an adenomatoid odontogenic tumor: report of a case with peculiar features J Oral Maxillofac Surg 1992 50 2 190 193 10.1016/0278-2391(92)90370-F 1732497

[B7] Cudney N Persico J Cordell KG D’Silva NJ Adenomatoid odontogenic tumor developing in association with an odontoma: report of a case Quintessence Int 2008 39 8 693 697 19107257

[B8] Reichart P Philipsen H Odontogenic tumors and allied lesions 2004 New Malden: Quintessence Publishing

[B9] Philipsen HP Reichart PA Adenomatoid odontogenic tumour: facts and figures Oral Oncol 1999 35 2 125 131 10.1016/S1368-8375(98)00111-0 10435145

[B10] Vargas PA Carlos-Bregni R Mosqueda-Taylor A Cuairan-Ruidiaz V Lopes MA de Almeida OP Adenomatoid dentinoma or adenomatoid odontogenic hamartoma: what is the better term to denominate this uncommon odontogenic lesion? Oral Dis 2006 12 2 200 203 10.1111/j.1601-0825.2005.01163.x 16476044

[B11] Otero D Israel MS Antero S Lourenco S Bilateral adenomatoid odontogenic hamartoma Oral Surg Oral Med Oral Pathol Oral Radiol Endod 2009 107 4 e24 e26 10.1016/j.tripleo.2008.12.045 19327632

[B12] Takeda Y Duct-like structures in odontogenic epithelium of compound odontoma J Oral Pathol Med 1991 20 4 184 186 10.1111/j.1600-0714.1991.tb00918.x 2061857

